# Challenges and Clinical Relevance in Diagnosing Metastatic Cells From Non‐Hematopoietic Malignancies in Bone Marrow Aspirates

**DOI:** 10.1002/cam4.70645

**Published:** 2025-02-05

**Authors:** Elise Kaspi, Charlotte Grosdidier, Yaël Berda‐Haddad, Maud Arpin, Sylvie Cointe, Shirley Fritz, Amandine Bonifay, Marie Koubi, Carine Jiguet‐Jiglaire, Patrice Roll, Diane Frankel

**Affiliations:** ^1^ Cell Biology Department, APHM, INSERM, MMG, Timone Hospital Aix Marseille Universite Marseille France; ^2^ Service of Medical Biology APHM, North Hospital Marseille France; ^3^ Medical Biological Laboratory—Hematology and Flow Cytometry Department Timone Hospital, APHM Marseille France; ^4^ Department of Internal Medicine CHU Nord, Assistance Publique‐Hôpitaux de Marseille (AP‐HM) Marseille France; ^5^ APHM, CNRS, INP, Inst Neurophysiopathol, GlioME Team, Réseau PrEclinique et TRAnslationnel de Recherche en Neuro‐Oncologie, CHU Timone, Service d'Anatomie Pathologique et de Neuropathologie Aix‐Marseille Universite Marseille France

**Keywords:** bone marrow aspirate, bone marrow aspiration, carcinoma, Ewing sarcoma, melanoma, metastasis

## Abstract

**Introduction:**

The causes of cytopenias are numerous, and the bone marrow aspirate helps to identify them. In rare cases, these cytopenias are due to bone marrow metastases from solid cancers. The techniques used in hematology laboratories are limited in characterizing these cells. Interaction with the cytopathology laboratory becomes critical for characterizing tumor cells and completing a comprehensive diagnosis from the bone marrow aspirate.

**Methods:**

This article describes a series of 38 bone marrow aspirates from 36 patients with bicytopenias who underwent bone marrow aspiration and for whom the hematologists sent the sample to the cytopathology laboratory to complete the diagnosis by immunocytochemistry and FISH if necessary.

**Results:**

The mean age of patients is 66 years, and the sex ratio is 2.8. Metastases were found in 11 cases of lung carcinoma, 4 cases of prostate carcinoma, 2 cases of breast carcinoma, 1 case of kidney carcinoma, 1 case of glioblastoma, 1 case of Ewing's sarcoma, and 1 case of melanoma. Among them, bone marrow aspiration was the only method to establish the initial diagnosis for seven patients. In six cases, immunocytochemistry confirmed the presence of carcinoma cells but could not identify their origin. In seven cases, tumor cells were insufficient to be characterized by immunocytochemistry.

**Conclusion:**

Collaboration between laboratories is essential for the management of bone marrow aspirates containing non‐hematopoietic metastases. Bone marrow aspiration may be sufficient to diagnose solid tumors, enabling faster initiation of treatment for patients already at an advanced stage of their disease.

## 1 Introduction

Bone marrow aspiration is a commonly performed procedure for the diagnosis and therapeutic monitoring of hematologic disorders. This minimally invasive procedure, which requires only local anesthesia, can be easily conducted during a routine medical consultation. Bone marrow aspirate provides both quantitative and qualitative information on hematopoietic cell populations, aiding in comprehensive clinical assessment. Standard laboratory techniques such as cytology, flow cytometry, and cytogenetic analysis are essential for the characterization and follow‐up of hematologic neoplasms [1]. Bone marrow aspiration is a common diagnostic tool for unexplained cytopenias, with hematologic malignancies being the primary underlying cause [2]. However, in a small subset of cases (1%–2%), bone marrow metastases may mimic a hematologic disorder and result in cytopenias. Bone marrow metastases occur in less than 10% of patients with metastatic cancers, with lung, breast, and prostate carcinoma exhibiting a higher prevalence [3]. The growth of metastatic carcinoma within the bone marrow leads to trabecular bone destruction, resulting in osteolytic lesions and subsequent cytopenias, predominantly anemia and thrombocytopenia.

Accurate identification of metastatic cells in bone marrow aspirates is critical for hematologists. While immunocytochemistry is essential for characterizing these cells, it is often not available in a hematology laboratory, as it is in a cytopathology laboratory. Therefore, effective collaboration between hematology and cytopathology laboratories is required to confirm the presence and nature of metastatic cells.

This article shows how such interdisciplinary collaboration, supported by cytological expertise, can significantly improve the detection of metastatic cells in bone marrow aspirates and improve the diagnostic management of patients at an advanced stage of their disease.

## 2 Methods

### 2.1 Sample Collection

This study included bone marrow aspirates received in the Hematology Laboratory of the North Hospital and La Timone Hospital (Assistance Publique des Hôpitaux de Marseille, France) from patients either with non‐hematologic cells or with newly diagnosed cytopenias in a context of known solid tumors. Smears and/or bone marrow aspirates collected in EDTA tubes were received. At least three smears were stained with May–Grünwald–Giemsa (MGG). When available, additional smears and EDTA samples were sent to the Cell Biology Laboratory at La Timone Hospital (Assistance Publique des Hôpitaux de Marseille, France) for further analysis.

### 2.2 Immunocytochemistry to Characterize Metastatic Cells

For cases where only smears were available, manual immunocytochemistry was performed until December 2023 using the SensiTEK HRP kit (ScyTek Laboratories, Logan, USA) on slides previously fixed in paraformaldehyde 4% for 10 min. Slides were incubated with peroxidase‐blocking solution for 30 min. After washing, slides were incubated with SensiTEK HRP kit for 10 min. Primary antibodies (Table 1) were incubated for 30 min. The biotinylated secondary antibodies were incubated for 15 min, followed by streptavidin/HRP for 20 min and DAB Quanto chromogen (Diagomics, Blagnac, France) for 5 min. Nuclei were counterstained with Mayer's hemalum solution. Slides were mounted with Aquatex.

**TABLE 1:** Antibodies used for immunocytochemistry to detect metastatic cells.

Antibody	Clone	Manufacturer	Dilution	Type
BerEP4	BerEP4	Dako	1/100	A
TTF1	8G7G3/1	Dako	1/100	A/M
P40	Polyclonal	Biocare Medical	1/100	M
P40	SP225	Roche	None	A
PAX8	MRQ‐50	Roche	None	A/M
GATA‐3	L50‐823	Roche	None	A/M
Pancytokeratin	Polyclonal	Ventana	None	M
AE1/AE3	Polyclonal	Roche	None	A
Epithelial Membrane Antigen	MOC31	Dako	1/50	M
CD45	2b11+PD7	Diagomics	1/500	A/M
PSA	35H9	Leica	1/100	A/M
Chromogranin	DAK‐A3	Dako	1/200	A/M
Synaptophysin	DAK‐SYNAP	Dako	1/100	A/M
CD56	MRQ‐42	Roche	None	A/M
CK7	OV.TL12/30	Dako	1/100	A/M
SOX10	EP268	MMF	1/100	A/M
NKX3.1	Polyclonal	Diagomics	1/100	A/M
NKX2.2	EP336	Diagomics	None	A

Abbreviations: A/M, Manual or automated technique; A, Automated technique on Benchmark Ultra; M, Manual technique using the SensiTEK HRP kit.

When bone marrow aspirate in EDTA tubes was available, smears or cytospins were performed after red cell lysis. In all cases, TOMO slides (Matsunami Glass, Bellingham, USA) were used to prevent the detachment of cells. Immunocytochemistry was performed on BenchMark ULTRA using the ultraview kit (Ventana, Roche, USA) from December 2023. The list of antibodies and the conditions used are provided in Table 1.

### 2.3 Flow Cytometry Analysis

Lymphocytes from bone marrow were analyzed by flow cytometry using the Navios 3‐laser instrument (Beckman Coulter, Miami, FL, USA). Fluorescent antibody reagents were procured from Beckman Coulter Immunotech (Marseille, France): CD45‐FITC (B3821F4A), CD3‐PC5 (UCHT1), CD4‐PE (SFCI12T4D11), CD8‐ECD (SFCI21Thy2D3), HLA‐DR‐PB (IMMU357), CD19‐ECD (J3.119), CD16‐PE (3G8), CD56‐PE (N901‐NKH1), and CD5‐PC7 (BL1a). Briefly, 100 μL of bone marrow was incubated with combinations of optimally titrated monoclonal antibodies for 15 min at room temperature in the dark. Finally, the samples were treated with a lysis buffer ([NH_₄_Cl] = 8.3 g/L), then washed with 2 mL PBS‐BSA1%‐azide, centrifuged at 300 *g* for 5 min, and resuspended in 500 μL BPS/BSA buffer. Data acquisition was conducted using Navios software, and the data were analyzed with Kaluza C software.

### 2.4 Fluorescence In Situ Hybridization

Smears were first washed with 2XSSC at 37°C for 2 min and then treated with pepsin‐HCl (0.001 g/L‐0.01 N) at 37°C for 13 min. After washing in PBS, post‐fixation was performed in formaldehyde‐MgCl2 (1% −0.05 M) for 5 min. Dehydration was carried out in baths containing increasing percentages of ethanol (70%, 85%, and 100%), and slides were air‐dried for 30 min. Smears were hybridized with the Vysis LSI EWSR1 Break Apart FISH Probe Kit (Abbott, IL, USA) for 17 h on Thermobrite (Leica, Nanterre, France). Post‐hybridization washes were performed in 0.4 SSC at 72°C for 2 min and 2 SSC‐0.01% Tween 20 at room temperature. Smears were air‐dried for 30 min before counterstaining with DAPI (Leica, Nanterre, France). Nuclei were observed with an Axio Imager Z2 microscope (Zeiss, France) and analyzed with Metafer software (Metasysems, Cormeilles‐en‐Parisis, France).

### 2.5 Consent

The project was reviewed and approved by the local ethics committee of treated with a lysisor their diagnosis and treatment. In accordance with the French data protection authority (Commission Nationale de l'Informatique et des Libertés), general information on research activities is provided in clinical units to departments and to the persons concerned, who may exercise their right to object, which is verified prior to data collection.

## 3 Results

The Cell Biology Laboratory analyzed 38 bone marrow aspirates from 36 patients to characterize metastatic cells in a known or unknown neoplastic context. The mean age of the patients was 66 years (range 21–90 years) and the sex ratio was 2.8.

**FIGURE 1:**
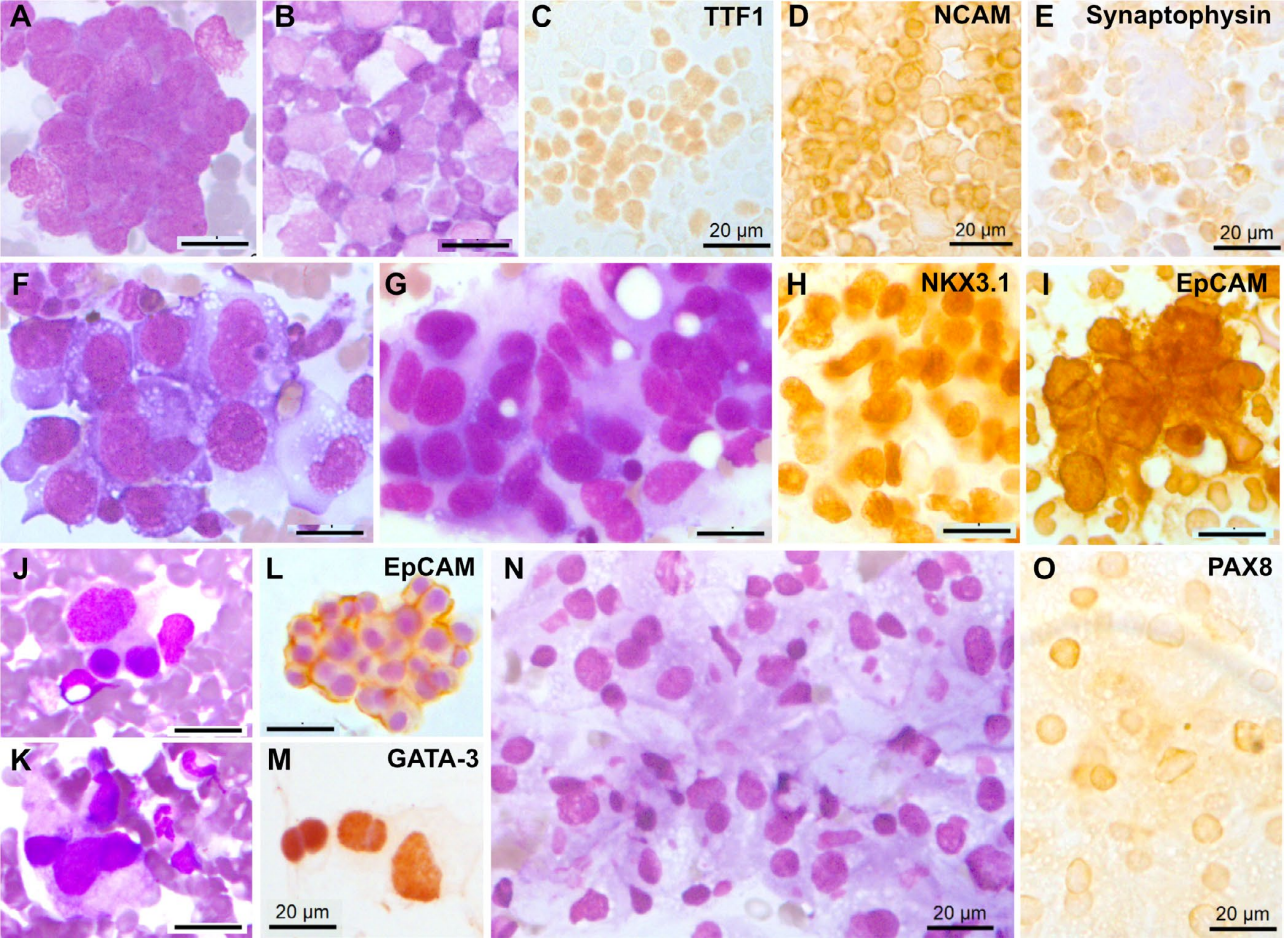
Carcinomas diagnosed from bone marrow aspirate with MGG staining and immunocytochemistry: Small cell lung carcinoma (SCLC) with May–Grünwald–Giemsa (MGG) staining on (A) smears and (B) cytospin slide obtained from EDTA sample. Immunocytochemistry confirming SCLC with the positivity of (C) TTF1, (D) NCAM/CD56, (E) synaptophysin. (F) Lung adenocarcinoma (MGG staining). Prostate carcinoma with MGG staining (G) confirmed by immunocytochemistry using (H) NKX3.1 and (I) EpCAM. Breast carcinoma with MGG staining (J and K) confirmed by immunocytochemistry using (L) EpCAM and (M) GATA‐3. Renal carcinoma with MGG staining (N) and confirmed with (O) PAX8 and EpCAM (not shown).

**FIGURE 2:**
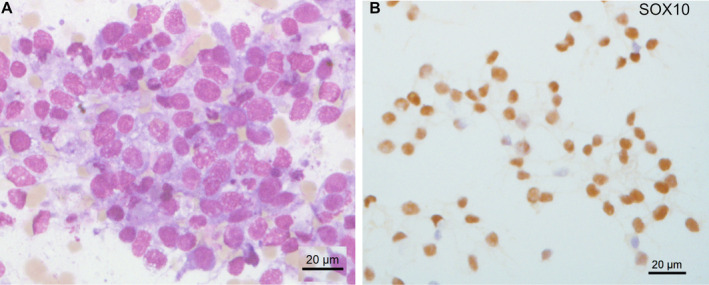
Melanoma diagnosed with MGG and immunocytochemistry. (A) MGG staining showing large sheets of tumor cells. (B) Immunocytochemistry showing nuclear positivity with SOX10.

**FIGURE 3:**
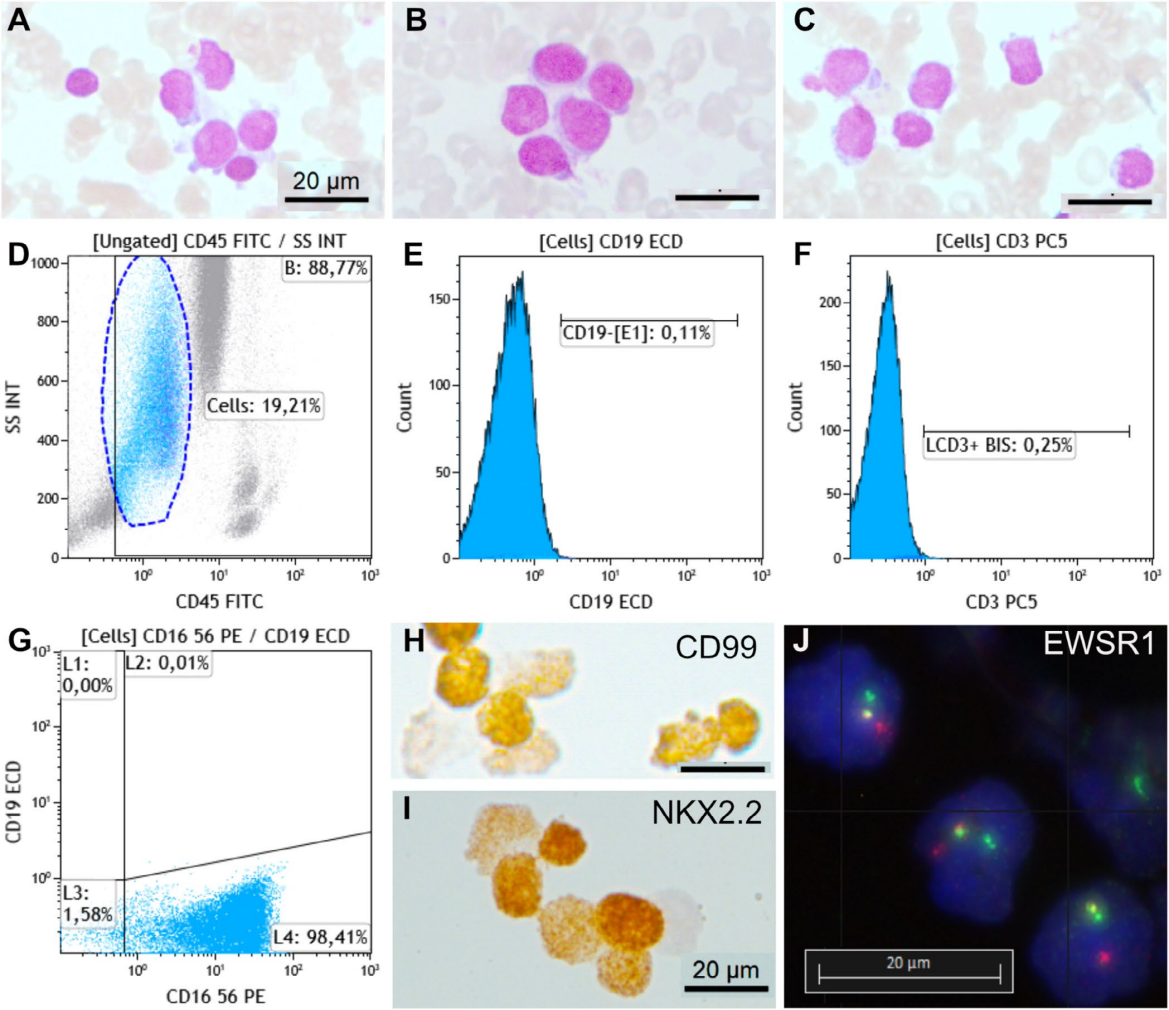
Ewing sarcoma. (A–C) Clusters or isolated small cells with a high nucleo‐cytoplasmic ratio (MGG staining). (D–G) Flow cytometry graph showing negative cells for CD45, CD19, and CD3 markers and positive cells for CD56. Immunocytochemistry showing positivity for (H) CD99 and (I) NKX2.2. (J) FISH with an EWSR1 break‐apart probe showing a separation of the red and green probes and confirming EWSR1 fusion.

Metastatic lung carcinoma was diagnosed in 11 cases (29%), including small cell lung cancer in 10 cases (Figure 1A–E) and lung adenocarcinoma in one case (Figure 1F). Prostate carcinoma was diagnosed in four cases (10.5%) (Figure 1G–I), breast carcinoma in two cases (5.2%) (Figure 1J–M), renal carcinoma (Figure 1N,O), melanoma (Figure 2), glioblastoma, and Ewing sarcoma (Figure 3) in one case each (2.6%). The last two cases were confirmed by bone marrow biopsy (Table 2). In 7 of these 21 cases, bone marrow aspirate was the only method or the fastest to establish the initial diagnosis. For example, in a 90‐year‐old patient, a prostate biopsy was not feasible due to high performance status. However, a bone marrow aspirate revealed metastatic prostate carcinoma, and genetic testing for BRCA1/2 was performed on the collected EDTA sample. Treatment was initiated based on the results of the bone marrow aspiration. A bone marrow aspirate from a 21‐year‐old patient showed small and round cells, with a high nucleo‐cytoplasmic ratio, often in clusters (Figure 3A–C). Flow cytometry analysis showed negativity for CD45, CD19, and CD3 markers but positivity for the CD56 marker (Figure 3D–G). Immunocytochemistry was positive for CD99 and NKX2.2 (Figure 3H,I). FISH analysis on the aspirate using an EWSR1 break‐apart probe confirmed the presence of an EWSR1 fusion, a hallmark of Ewing sarcoma (Figure 3J). Bone marrow aspiration results were obtained 1 week earlier than those from a bone marrow biopsy, allowing for earlier initiation of treatment.

**TABLE 2:** Results of the 38 bone marrow aspirations from 36 patients.

Patient (number of bone marrow aspirates)	Age (years old)	Sex	Diagnosis on bone marrow aspiration	Diagnosis on biopsy (type and date)	Death (time elapsed after the bone marrow aspiration)
1	69	M	Lung adenocarcinoma	Lung adenocarcinoma (liver biopsy, 4 months before)	Yes (11 days)
2	58	M	SCLC	SCLC (left inferior lung biopsy, same date)	No
3	78	M	SCLC	None	Yes (9 days)
4	73	M	SCLC	SCLC (upper right lung biopsy, same date)	Not known
5	65	M	SCLC	None	Yes (13 days)
6	62	M	SCLC	SCLC (left mediastinal‐hilar biopsy, 4 days before)	Not known
7	75	M	SCLC	SCLC (lymph node 7 biopsy, same date)	Yes (25 days)
8	52	F	SCLC	None (CT scan reveals a lung mass with metastases)	Not known
9	63	M	SCLC	SCLC (left carina biopsy, same date)	Yes (14 days)
10	63	M	SCLC	None	Yes (6 months)
11	54	M	SCLC	None (SCLC confirmed on right clavicle biopsy, 2 days later)	No
12	73	M	Prostatic carcinoma	Prostatic carcinoma (prostate,2 years before)	No
13	76	M	Prostatic carcinoma	Prostatic carcinoma (prostate, 15 years before with a relapse 5 years before)	No
14	87	M	Prostatic carcinoma	Prostatic carcinoma (prostate, 7 years before)	Not known
15	90	M	Prostatic carcinoma	None, BRCA testing performed on the bone marrow aspirate	Yes (18 days)
16	86	F	Breast carcinoma	Breast carcinoma (right breast biopsy, 1 month before)	No
17	63	F	Breast carcinoma	Breast carcinoma (breast biopsy, 3 years before)	Not known
18	21	M	Ewing sarcoma	None (bone marrow biopsy confirmed the diagnosis 1 week after)	Not known
19	76	F	Glioblastoma	Glioblastoma (left temporal biopsy, 6 months before)	No
20	34	M	Melanoma	Melanoma (left inguinal lymph node, 1 month before)	(Yes 2 days)
21	58	M	Renal cell carcinoma	Renal cells carcinoma (left iliac mass, 7 years before; kidney, 11 years before)	Not known
22	70	F	Carcinoma	Breast carcinoma (mastectomy, 13 years before with a relapse 3 years before)	
23	89	M	Carcinoma	Renal cells carcinoma (date of diagnosis not known)	Not known
24	51	M	Carcinoma	None (CT scan reveals a mass developed at the expense of the gastric wall)	Yes (11 days)
25 (2)	67	F	Carcinoma	Breast carcinoma (bone marrow biopsy, same date)	No
26	77	M	Carcinoma	None (prostate biopsy confirmed prostatic carcinoma 1 month later)	Yes (4 months)
27	68	F	None (too few metastatic cells)	Pancreatic carcinoma (gastric biopsy, 1 month before)	Yes (1 month)
28 (2)	66	M	None (too few metastatic cells)	History of melanoma. Prostatic carcinoma diagnosed on bone marrow biopsy 3 months later	No
29	69	M	None (too few metastatic cells)	None (prostatic carcinoma confirmed on prostate biopsy 3 weeks later)	Not known
30	56	M	None (too few metastatic cells)	History of cutaneous T‐cell lymphoma	Not known
31	82	M	None (too few metastatic cells)	None (renal cells carcinoma confirmed on right iliac wing 2 months later)	Yes (2 months)
32	39	M	None (too few metastatic cells)	None (medulloblastoma confirmed on left femur biopsy 1 month later)	Not known
33	74	M	Absence of metastatic cells	Lung adenocarcinoma (lung biopsy, 2 years before)	Yes (22 days)
34	54	M	Absence of metastatic cells	Squamous lung cancer (lung biopsy, 4 years before)	Not known
35	88	F	Absence of metastatic cells	Renal cell carcinoma (lung biopsy, 3 months before)	Yes (4 months)
36	58	F	Absence of metastatic cells	Lung adenocarcinoma (brain biopsy, 3 years before)	Not known

Abbreviation: SCLC, small cell lung cancer.

In six other bone marrow aspirates from five patients, immunocytochemistry revealed metastatic carcinoma cells positive for cytokeratins and BerEP4/EpCAM. Four of these patients had previously been diagnosed with a carcinoma of known origin (2 breast, 1 prostate, and 1 renal). However, immunocytochemistry failed to identify these possible origins based on the known primitive tumor (negative for GATA‐3, NKX3.1, or PAX8 respectively) or to identify other origins. In the remaining case, the bone marrow aspirate was the initial evidence of carcinoma, and a subsequent chest tomography scan revealed a mass that had developed at the expense of the gastric wall. Unfortunately, the patient died 11 days after the bone marrow aspiration without biopsy confirmation.

In seven cases, cells suspicious of non‐hematopoietic neoplasia were observed on the smears. However, immunocytochemical analyses were inconclusive due to the insufficient number of these cells. Among them, a patient with melanoma undergoing immunotherapy presented with bicytopenia. The challenge was to differentiate between a bicytopenia due to metastasis and a side effect of the immunotherapy. Two consecutive bone marrow aspirations were performed, and metastatic cells were detected in limited numbers only in the first aspirate. However, the subsequent bone marrow biopsy performed several weeks later revealed metastatic prostate cancer in this patient.

In four cases with a history of solid cancers (lung adenocarcinoma, small bowel neuroendocrine tumor, renal cell carcinoma) and bicytopenias, the bone marrow aspirate was sent by the hematologists to the cell biology laboratory to rule out metastatic disease, which was done.

Fourteen patients died shortly after the bone marrow aspiration (median 20 days, range 2 days to 6 months). The earliest death occurred in a 34‐year‐old patient who had been diagnosed with melanoma 1 month earlier (Figure 2). Among the 11 patients with lung carcinoma, 4 died within 15 days of the bone marrow aspiration, 1 died within a month, 1 died within 6 months, 3 patients were lost to follow‐up, and 2 were alive 1 year and 4 years after the bone marrow aspiration.

## 4 Discussion

The diagnosis of non‐hematopoietic metastatic cells in a bone marrow sample requires the identification of cells that do not belong to the normal hematologic lineage. This relies on the expertise of hematologists. Flow cytometry can confirm their non‐hematopoietic origin by identifying cells lacking CD45 expression. The cytologic characteristics of non‐hematopoietic metastatic cells vary depending on the type of tumor. Carcinomas often occur in clusters, making them easier to detect. Small cell lung cancer, neuroblastoma, retinoblastoma, and Ewing sarcoma present as small, round cells with high nucleo‐cytoplasmic ratios, often in clusters. Recently, artificial intelligence tools are being developed to assist pathologists and hematologists in diagnosing metastatic tumor cells in bone marrow aspirates [4].

In the context of non‐hematopoietic metastases, immunocytochemistry is essential for the characterization of cancer cells, especially those of unknown primary origin. Optimization of both the material and the technique used is essential for accurate diagnosis [5]. Our laboratory has improved immunocytochemistry by moving to an automated method for both smears and cytospins from EDTA tubes. Positive‐charged slides are critical to prevent cell detachment during automated procedures. This technical issue has led to several failures in cell characterization. Cytoblocks can also be prepared from EDTA tubes if available for immunohistochemical characterization. In our experience, bone marrow aspirates in the EDTA tubes are ideal because they allow for the preparation of smears, cytospins, and cytoblocks, and thus can be adapted to various laboratory techniques and allow for genetic analysis. Receiving smears directly from clinical services limits the number of antibodies that can be tested because of the limited number of smears available. The main challenge in characterizing metastatic cancer cells is the insufficiency of neoplastic cells in the samples. For some patients, cells were visible on only a few smears and often absent from smears used for immunochemistry, making characterization difficult. While bone marrow biopsy is the preferred method for characterizing non‐hematopoietic cells, it requires general anesthesia, which is not always feasible in patients with severe thrombocytopenia, and has a longer turnaround time due to decalcification. Bone marrow aspirates provide faster results, help start chemotherapy on time, and complement biopsies when they are inconclusive. However, bone marrow biopsy still remains more effective than bone marrow aspirate in detecting metastasis [6, 7].

Our study mainly included patients with metastatic small cell lung carcinoma, whereas other studies reported a predominance of gastric tumors [4, 8]. These differences may be attributed to the recruitment practices of each hospital. The Nord Hospital in Marseille has a very active Oncopneumology Department, which could explain the overrepresentation of small cell neuroendocrine carcinoma cases in our study compared to other centers.

In our series, we diagnosed a case of Ewing sarcoma confirmed by EWSR1 fusion by FISH. Flow cytometry confirmed the non‐hematopoietic origin of the metastatic cells by negative expression of CD45, CD19, and CD3, despite CD56 positivity. In a study of 46 Ewing sarcomas, CD56 positivity was found in 60% and was significantly associated with progression‐free survival [9]. The patients underwent a bone marrow biopsy, confirming these findings. Ewing sarcoma is the second most common bone tumor in children and adolescents. Bone marrow is a common site of metastasis (5%–17%) after bone (30%–40%) and lung (50%) [10]. The National Ewing Sarcoma Tumor Board recommended in 2023 that if imaging suggests metastatic involvement of the bone marrow at diagnosis, bilateral bone marrow biopsies and bone marrow aspiration should be performed to confirm the involvement and to establish a baseline for evaluating therapeutic response [11].

Patients with bone marrow metastases have a poor prognosis due to rapid disease progression and limited response to treatment [12, 13]. In our series, 39% of patients died within days or weeks after puncture. Kucukzeybek et al. [8] reported differences in survival depending on the origin of the carcinoma between breast and gastric carcinomas. In our series of lung carcinoma, the very short mean overall survival of 14 days after puncture is probably linked to the aggressiveness of small cell lung cancer. The poor survival of patients in our cohort is also due to the selection of patients requiring a bone marrow aspiration. Indeed, bone marrow aspiration is performed in patients known to have solid tumors, either when there is an indication for a change in therapeutic line or in cases of suspected drug toxicity induced by chemotherapy or immunotherapy. These patients are typically at a more advanced stage of their disease. There are also patients for whom bone marrow metastases are discovered incidentally because the cancer was previously unknown. These patients are therefore diagnosed at an already advanced stage of their disease, and the bone marrow biopsy becomes advantageous for diagnosis. It can also be used for molecular biology analyses, which are often necessary before the initiation of chemotherapy.

## 5 Conclusion

Collaboration between hematologic and non‐hematologic cytologists is critical to the management of metastatic bone marrow aspirates. In our experience, the techniques used by the cell biology laboratory allow diagnosis without the need for additional samples or biopsies. For these patients, who have a short average survival, obtaining reliable and timely results is essential for initiating or adjusting treatment.

## Author Contributions

**Elise Kaspi:** conceptualization, writing – review and editing, methodology, formal analysis. **Charlotte Grosdidier:** formal analysis, writing – review and editing, resources. **Yaël Berda‐Haddad:** formal analysis, writing – review and editing, resources. **Maud Arpin:** formal analysis, writing – review and editing, resources. **Sylvie Cointe:** resources, formal analysis, writing – review and editing. **Shirley Fritz:** resources, formal analysis, writing – review and editing. **Amandine Bonifay:** resources, formal analysis, writing – review and editing. **Marie Koubi:** resources, formal analysis, writing – review and editing. **Carine Jiguet‐Jiglaire:** resources, formal analysis, writing – review and editing. **Patrice Roll:** resources, formal analysis, writing – review and editing. **Diane Frankel:** conceptualization, investigation, writing – original draft, methodology, validation, formal analysis, resources, supervision.

## Ethics Statement

The project has been reviewed and approved by the local ethics committee of Assistance Publique‐Hôpitaux de Marseille (approval number n°WQ4C73). The data for this study were obtained from patients treated at Assistance Publique‐Hôpitaux de Marseille for their diagnosis and treatment. In accordance with the French data protection authority (Commission Nationale de l'Informatique et des Libertés), general information on research activities is provided in clinical units to departments to the persons concerned, who may exercise their right to object, which is verified prior to data collection. This procedure exempts the written informed consent for all patients as granted by the local ethics committee of Assistance Publique‐Hôpitaux de Marseille.

## Conflicts of Interest

The authors declare no conflicts of interest.

## Data Availability

Data sharing not applicable to this article as no datasets were generated or analyzed during the current study.
